# Mitogenome of northern long-eared bat

**DOI:** 10.1080/23802359.2020.1830726

**Published:** 2020-10-21

**Authors:** Sarah J. Gaughan, Kevin L. Pope, Jeremy A. White, Cliff A. Lemen, Patricia W. Freeman

**Affiliations:** a Bellevue University, Bellevue, NE, USA; b Nebraska Cooperative Fish and Wildlife Research Unit, and School of Natural Resources, University of Nebraska-Lincoln, Lincoln, NE, USA; c U.S. Geological Survey—Nebraska Cooperative Fish and Wildlife Research Unit, and School of Natural Resources, University of Nebraska-Lincoln, Lincoln, NE, USA; d Department of Biology, University of Nebraska Omaha, Omaha, NE, USA; e University of Nebraska-Lincoln, Lincoln, NE, USA; f School of Natural Resources, and University of Nebraska State Museum, University of Nebraska-Lincoln, Lincoln, NE, USA

**Keywords:** *Myotis septentrionalis*, mitochondrial genome, next generation sequencing

## Abstract

The complete mitogenome of the northern long-eared bat (*Myotis septentrionalis)* was determined to be 17,362 bp and contained 22 tRNA genes, 2 rRNA genes and one control region. The whole genome base composition was 33.8% GC. Phylogenetic analysis suggests that *M. septentrionalis* be positioned next to *M. auriculus* in the Nearctic subclade of the *Myotis* genus. This complete mitochondrial genome provides essential molecular markers for resolving phylogeny and future conservation efforts.

The northern long-eared bat (*Myotis septentrionalis*) has recently experienced drastic population declines in eastern and midwestern parts of its range because of the invasive fungal disease white-nose syndrome (WNS) (Frick et al. 2015; Langwig et al. 2015). The disease induces physiological and behavioral changes in bats during hibernation, which can result in death (Verant et al. 2014). Population declines have been so severe that *M. septentrionalis* was listed as threatened in the United States of America (USFWS 2015) and endangered in Canada (COSEWIC 2013). *M. septentrionalis* seems to be more susceptible to WNS than other closely related species, such as the little brown bat (*Myotis lucifugus*); however, the cause of this susceptibility has yet to be determined and may be due to genetic differences or varying environmental preferences (Frick et al. 2015; Langwig et al. 2016). Regulation of specific mitochondrial genes, including *COI*, *ND2*, *ATP6* and *ATP8*, is crucial during the hibernation process (Hittel and Storey 2002); therefore, comparative analysis of mitochondrial genomes of hibernating bat species might offer some insight into how WNS affects species differently. Here we report the first complete mitogenome of *M. septentrionalis* and examine the phylogenetic position of *M. septentrionalis* within the genus *Myotis* based on complete mitogenomes.

We collected wing tissue from an adult, female *M. septentrionalis* on 5 July 2017 at Ponca State Park (Dixon County) in northeastern Nebraska (42.6022° N, 96.7154° W). We used sterile 2-mm disposable biopsy punches to collect two tissue plugs from the flight membrane near the leg of the bat to minimize effects on future flight performance. Care was taken to avoid large blood vessels, which were easily seen in the flight membrane. A representative tissue plug from an adult, female *M. septentrionalis* that was collected at the same site on the same evening was deposited at the University of Nebraska State Museum (catalog number UNSM ZM-31046). After tissue collection, bats were released at points of capture. Each tissue plug was stored dry in a cryogenic tube with several silica beads. Upon return from the field, tissue plugs were frozen at −80 °C until DNA extraction. Genomic mitochondrial DNA was extracted and purified from one tissue plug using the standard protocol of the Abcam Mitochondrial DNA Isolation Kit and sequenced on an Illumina NextSeq500 at the University of Nebraska Medical Center. The mitogenomic sequence was assembled and annotated using Geneious (Kearse et al. 2012).

The total length of the mitogenome was 17,362 bp (GenBank Accession No. MK547202). The mitogenome consisted of 22 tRNA genes, two rRNA genes and one control region. The whole genome base composition was 33.8% GC.

To investigate the position of *M*. *septentrionalis* within the genus *Myotis*, we constructed a maximum likelihood tree based on 19 complete mitochondrial genomes using MEGA 6 under the GTR + G + I model with 500 bootstrap replicates (Pattengale et al. 2010; Tamura et al. 2013). The phylogenetic tree contained two subclades, Nearctic and Neotropical (Figure 1). The position of *M*. *septentrionalis* next to *M. auriculus* in the Nearctic subclade of the *Myotis* genus corresponds with previously proposed phylogenetic relationships (Stadelmann et al. 2007).

**Figure 1.**
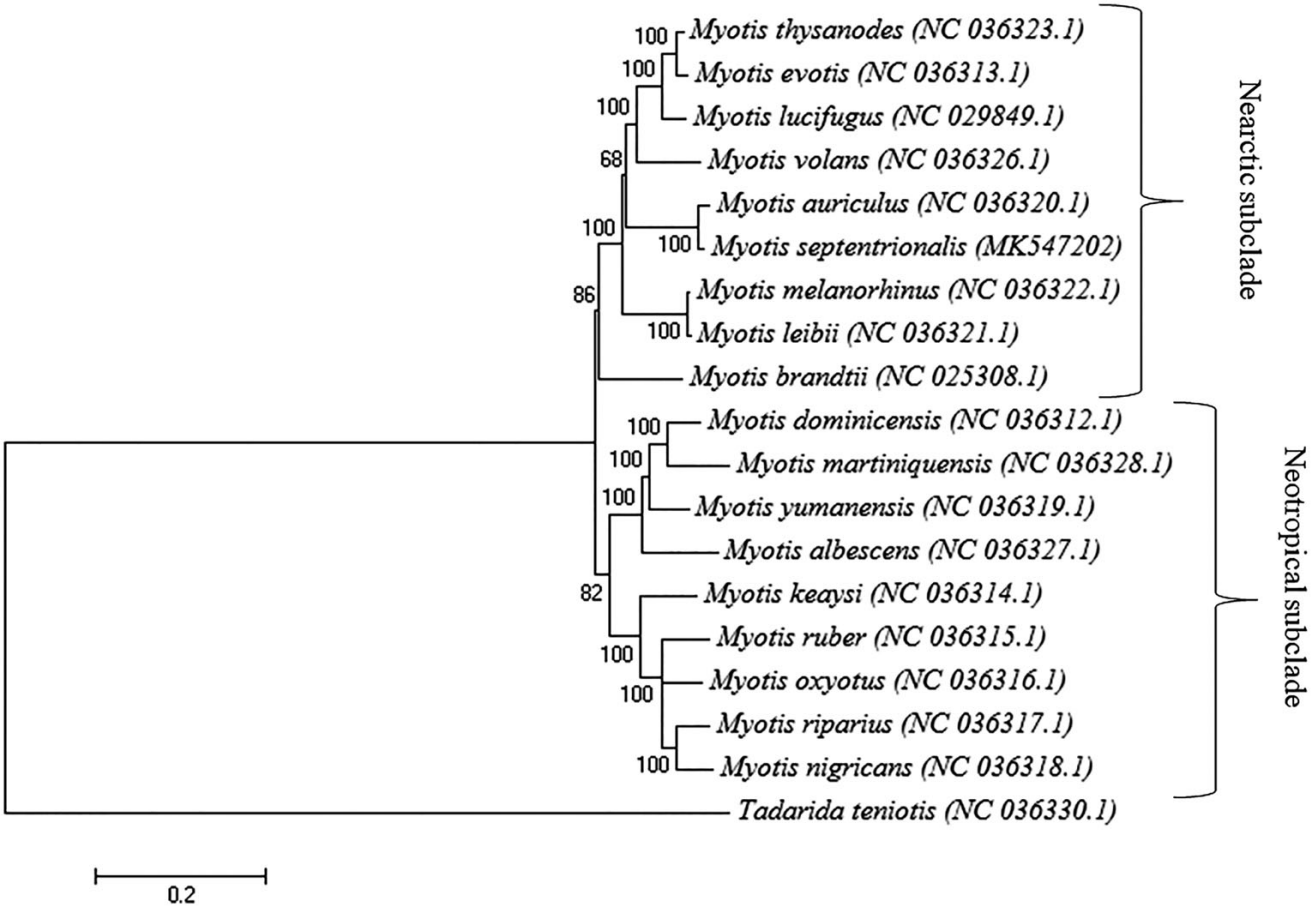
Phylogenetic tree generated using a maximum likelihood method and a general time reversal model based on nineteen complete mitochondrial genomes. The GenBank accession number is listed next to each species within the tree.

This mitogenome establishes a basis for additional, future phylogenetic studies of this diverse genus, as well as studies on the effects of WNS on *M. septentrionalis* in comparison to other species of hibernating bats. Future studies should consider the susceptibility of *Myotis* bats to WNS in relation to their Nearctic and Neotropical subclade groupings.

## Data Availability

The data that support the findings of this study are openly available in GenBank of NCBI at https://www.ncbi.nlm.nih.gov, reference number MK547202.

## References

[CIT0001] COSEWIC 2013. COSEWIC assessment and status report on the Little Brown Myotis Myotis lucifugus, Northern Myotis Myotis septentrionalis and Tri-colored Bat Perimyotis subflavus in Canada. Committee on the Status of Endangered Wildlife in Canada xxiv + 93 pp.

[CIT0002] Frick WF, Puechmaille SJ, Hoyt JR, Nickel BA, Langwig KE, Foster JT, Barlow KE, Bartonička T, Feller D, Haarsma A-J, et al. 2015. Disease alters macroecological patterns of North American bats. Global Ecol Biogeogr. 24(7):741–749.

[CIT0003] Hittel DS, Storey KB. 2002. Differential expression of mitochondria-encoded genes in a hibernating mammal. J Exp Biol. 205:1625–1631. 12000807 10.1242/jeb.205.11.1625

[CIT0004] Kearse M, Moir R, Wilson A, Stones-Havas S, Cheung M, Sturrock S, Buxton S, Cooper A, Markowitz S, Duran C, et al. 2012. Geneious basic: an integrated and extendable desktop software platform for the organization and analysis of sequence data. Bioinformatics. 28(12):1647–1649. 22543367 10.1093/bioinformatics/bts199 PMC3371832

[CIT0005] Langwig KE, Frick WF, Hoyt JR, Parise KL, Drees KP, Kunz TH, Foster JT, Kilpatrick AM. 2016. Drivers of variation in species impacts for a multi-host fungal disease of bats. Phil Trans R Soc B. 371(1709):20150456. 28080982 10.1098/rstb.2015.0456 PMC5095535

[CIT0006] Langwig KE, Hoyt JR, Parise KL, Kath J, Kirk D, Frick WF, Foster JT, Kilpatrick AM. 2015. Invasion dynamics of white-nose syndrome fungus, midwestern United States, 2012–2014. Emerging Infect Dis. 21(6):1023–1026. 10.3201/eid2106.150123 PMC4451901 25989230

[CIT0007] Pattengale ND, Alipour M, Bininda-Emonds ORP, Moret BME, Stamatakis A. 2010. How many bootstrap replicates are necessary? J Comput Biol. 17(3):337–354. 20377449 10.1089/cmb.2009.0179

[CIT0008] Stadelmann B, Lin L-K, Kunz TH, Ruedi M. 2007. Molecular phylogeny of New World *Myotis* (Chiroptera, Vespertilionidae) inferred from mitochondrial and nuclear DNA genes. Mol Phylogenet Evol. 43(1):32–48. 17049280 10.1016/j.ympev.2006.06.019

[CIT0009] Tamura K, Stecher G, Peterson D, Filipski A, Kumar S. 2013. MEGA6: molecular evolutionary genetics analysis version 6.0. Mol Biol Evol. 30(12):2725–2729. 24132122 10.1093/molbev/mst197 PMC3840312

[CIT0010] USFWS. 2015. Endangered and threatened wildlife and plants; threatened species status for the Northern Long-eared Bat with 4(d) rule. Fed Regist. 80(63):17974–18033.

[CIT0011] Verant ML, Meteyer CU, Speakman JR, Cryan PM, Lorch JM, Blehert DS. 2014. White-nose syndrome initiates a cascade of physiologic disturbances in the hibernating bat host. BMC Physiol. 14:10. 25487871 10.1186/s12899-014-0010-4 PMC4278231

